# Detection and Genomic Characterization of Enterovirus D68 in Respiratory Samples Isolated in the United States in 2016

**DOI:** 10.1128/genomeA.01350-16

**Published:** 2016-12-08

**Authors:** Terry Fei Fan Ng, Anna Montmayeur, Christina Castro, Marshall Cone, Joey Stringer, Daryl M. Lamson, Shannon L. Rogers, Shur-Wern Wang Chern, Laura Magaña, Rachel Marine, Heather Rubino, Daniel Serinaldi, Kirsten St. George, W. Allan Nix

**Affiliations:** a Division of Viral Diseases, Centers for Disease Control and Prevention, Atlanta, Georgia, USA; b CSRA International, Atlanta, Georgia, USA; c Oak Ridge Institute for Science and Education, Oak Ridge, Tennessee, USA; d Florida Department of Health, Tallahassee, Florida, USA; e Dallas County Health and Human Services, Dallas, Texas, USA; f Wadsworth Center, New York State Department of Health, Albany, New York, USA

## Abstract

The genomic sequences of three 2016 enterovirus D68 (EV-D68) strains were obtained from respiratory samples of patients from Florida, Texas, and New York. These EV-D68 sequences share highest nucleotide identities with strains that circulated in North America, Europe, and Asia in 2014–2015.

## GENOME ANNOUNCEMENT

Enterovirus D68 (EV-D68) (genus *Enterovirus*, family *Picornaviridae*) can cause severe respiratory illness, with clinical manifestations that include bronchiolitis, wheezing, and pneumonia, especially in children (1). The large 2014 outbreak of EV-D68 respiratory disease in the United States was caused by two co-circulating strains of EV-D68, with a predominant strain accounting for >91% of laboratory-confirmed cases (1, 2). A cluster of severe neurological disease, clinically characterized as acute flaccid myelitis (AFM), was temporally associated with the 2014 respiratory disease outbreak; however, no direct laboratory evidence conclusively linking EV-D68 with AFM was obtained (3). CDC initiated surveillance for AFM during the 2014 outbreak to help further define the incidence, epidemiology, and etiology of AFM. Twenty-one AFM cases were reported to CDC in 2015; none of the 2015 AFM clinical specimens tested were positive for EV-D68 (http://www.cdc.gov/acute-flaccid-myelitis/afm-surveillance.html).

Suspected AFM cases were reported to CDC by public health laboratories in Florida, Texas, and New York in the spring of 2016, with positive EV-D68 detections in nasopharyngeal swabs (NP) from all three patients, using EV-D68-specific real-time reverse transcription-PCR (RT-PCR) (http://www.fda.gov/MedicalDevices/Safety/EmergencySituations/ucm161496.htm). Two patients were subsequently confirmed as having AFM (FL and TX) and one patient with limb weakness did not meet the full case definition. To obtain the complete genome sequences of EV-D68 in the NP samples, a next-generation sequencing (NGS) approach was employed. RNA was extracted from the NP in viral transport medium, using QIAmp Viral RNA extraction kits (Qiagen). Three sets of overlapping consensus primers covering the 5′ NTR-VP1, VP1−2C, and 2C-3′NTR regions (TTAAAACAGCYTTGGGGTTGT [1 to 21] and GCTGATTTATCACTGTGCGAGTT [2544 to 2522]; AACTTGGTGTGGTCCCTAGC [2446 to 2465] and CCTTTCTTTTGGCAATAATCC [5134 to 5114]; and TCTCAAGAAGTTAGGGATTATTGC [5100 to 5123] and GGCCCCCAAGTGACCAAAAT [7333 to 7314] [coordinates per GenBank KT285485]) were used to amplify the genome of EV-D68. Three RT-PCRs were performed, using the One-Step RT-PCR Kit (Qiagen) on each RNA sample with a “touch-down” protocol (4). For each sample the three amplicons were pooled, purified, and subjected to Nextera XT (Illumina) library preparation, followed by sequencing using the Illumina MiSeq platform. A custom bioinformatics pipeline was used to assemble the genomes (5, 6).

The 3 EV-D68 sequences, US/FL/2016-19504, US/TX/2016-19506, and US/NY/2016-19505, varied from 7,311 to 7,333 nucleotides (nt) in length, but all contained the entire polyprotein of 6,567 nt. The three United States 2016 strains shared 98.6% to 99.7% nt identity (nt id) and 99.7% to 99.8% amino-acid identity (aa id) in the polyprotein; they shared 98.0% to 99.4% nt id and 99.0% to 99.4% aa id in the VP1 capsid region.

Based on scant EV-D68 VP1 nucleotide sequence data in GenBank, the 2016 United States strain is most closely related to EV-D68 strains from China in 2015 (KU982558 to KU982559; 97.8% to 98.6% nt id, 98.4% to 99.4% aa id) and strains identified in fall 2014 in Germany (KP657745; 98.1% to 98.3% nt id, 98.7% to 99.0% aa id) and in British Columbia, Canada (KT873552 to KT873553; 97.8% to 98.1% nt id, 98.4% to 99.6% aa id; incomplete VP1, missing 3′ end). The 2016 EV-D68 strain shares 95.0% to 95.1% nt id and 96.8% to 97.4% aa id with the predominant 2014 outbreak strain from the United States (KM851227) (1). The three EV-D68 genomes from this study constitute a single strain on an evolutionary continuum from 2014, showing divergence typical of circulating enteroviruses over time.

### Accession number(s).

The sequences of US/FL/2016-19504, US/TX/2016-19506, and US/NY/2016-19505 have been deposited in GenBank under accession numbers KX675261 to KX675263, respectively.
